# Effect of Speech Recognition on Problem Solving and Recall in Consumer Digital Health Tasks: Controlled Laboratory Experiment

**DOI:** 10.2196/14827

**Published:** 2020-06-01

**Authors:** Jessica Chen, David Lyell, Liliana Laranjo, Farah Magrabi

**Affiliations:** 1 Centre for Health Informatics Australian Institute of Health Innovation Macquarie University North Ryde Australia

**Keywords:** speech recognition software, consumer health informatics, ergonomics

## Abstract

**Background:**

Recent advances in natural language processing and artificial intelligence have led to widespread adoption of speech recognition technologies. In consumer health applications, speech recognition is usually applied to support interactions with conversational agents for data collection, decision support, and patient monitoring. However, little is known about the use of speech recognition in consumer health applications and few studies have evaluated the efficacy of conversational agents in the hands of consumers. In other consumer-facing tools, cognitive load has been observed to be an important factor affecting the use of speech recognition technologies in tasks involving problem solving and recall. Users find it more difficult to think and speak at the same time when compared to typing, pointing, and clicking. However, the effects of speech recognition on cognitive load when performing health tasks has not yet been explored.

**Objective:**

The aim of this study was to evaluate the use of speech recognition for documentation in consumer digital health tasks involving problem solving and recall.

**Methods:**

Fifty university staff and students were recruited to undertake four documentation tasks with a simulated conversational agent in a computer laboratory. The tasks varied in complexity determined by the amount of problem solving and recall required (simple and complex) and the input modality (speech recognition vs keyboard and mouse). Cognitive load, task completion time, error rate, and usability were measured.

**Results:**

Compared to using a keyboard and mouse, speech recognition significantly increased the cognitive load for complex tasks (*Z*=–4.08, *P*<.001) and simple tasks (*Z*=–2.24, *P*=.03). Complex tasks took significantly longer to complete (*Z*=–2.52, *P*=.01) and speech recognition was found to be overall less usable than a keyboard and mouse (*Z*=–3.30, *P*=.001). However, there was no effect on errors.

**Conclusions:**

Use of a keyboard and mouse was preferable to speech recognition for complex tasks involving problem solving and recall. Further studies using a broader variety of consumer digital health tasks of varying complexity are needed to investigate the contexts in which use of speech recognition is most appropriate. The effects of cognitive load on task performance and its significance also need to be investigated.

## Introduction

Recent advances in natural language processing and artificial intelligence have led to improvements in and widespread adoption of speech recognition technologies [1]. Speech recognition is an input modality that translates human speech into computerized text [2]. In consumer applications, speech recognition is usually applied as a way to interact with conversational agents, which are systems that mimic human conversation using text or spoken language [3,4]. Consumer conversational agents (such as Amazon Alexa and Google Assistant) can improve patient workflow by allowing patients to call nurses [5]. In health care, conversational agents have been utilized for a variety of purposes, including data collection, decision support, and patient monitoring [5-8]. 

Problems with the use of digital health technology represent a well-documented safety concern in the literature [9,10]. However, little is known about the problems associated with conversational agents that pose actual or potential risks of harm to consumers [9]. One study in which the participants were asked to interact with conversational agents identified significant safety concerns arising from the quality of information provided in response to health-related questions [11]. For example, incorrect information provided by a conversational agent in response to a question about the amount of alcohol that could be consumed while taking oxycodone could lead to severe harm, including death. However, few studies have evaluated conversational agents in the hands of consumers [3,4].

In other consumer-facing tools, cognitive load has been observed to be an important factor affecting the use of speech recognition technologies. Users reported finding it more difficult to think and speak at the same time when compared to typing, pointing, and clicking [12,13]. However, the effects of speech recognition use on cognitive load when performing health tasks has not yet been explored. Cognitive load is the amount of workload imposed on the brain’s working or short-term memory, which has limited capacity [14,15] and a short duration [16], particularly when performing tasks requiring problem solving and recall [14,17]. An example of a problem-solving task is using basic arithmetic to calculate nutritional information, whereas recall involves memorizing and reporting exercise information. Due to the cognitive load, certain tasks may be more difficult to perform because speaking shares the same cognitive resources in the brain as those required for problem solving and recall (ie, working memory) [12]. Therefore, a possible challenge with the use of speech recognition is that it can increase the cognitive load in tasks requiring more problem solving and recall.

Although studies in other domains have investigated the effects of speech recognition use on cognitive load, to our knowledge, no study has measured its effects in digital health tasks [12,18,19]. Thus, the aim of the present study was to evaluate the use of speech recognition for documentation in consumer digital health tasks such as recording diet and exercise information in comparison to using a conventional keyboard and mouse. The following hypotheses were tested: (1) cognitive load is higher for speech recognition compared to keyboard and mouse use in complex tasks requiring more problem solving and recall; (2) the percentage of errors is higher for speech recognition compared with keyboard and mouse use in complex tasks requiring more problem solving and recall; (3) task completion time is lower for speech recognition compared with keyboard and mouse use in simple tasks requiring less problem solving and recall; and (4) speech recognition is less usable than a keyboard and mouse for both simple and complex tasks requiring more problem solving and recall.

These findings will shed light on the characteristics of consumer digital health tasks that make them most suitable for using speech recognition as an input modality.

## Methods

### Participants

Fifty-two university students and staff participated in this study (see Results for a summary of the participant demographics). The participants were either students or staff who met the minimum English language proficiency for admission to a university program or workplace. These participants also had working knowledge of computer technology and systems as required for their degree or profession. Hence, there were no requirements regarding level of English language, health literacy, or technology proficiency for inclusion. The participants responded to advertisements sent by email or published in a university newsletter. Consenting adults aged 18 years or older were eligible to participate. Ethical approval was granted by Macquarie University’s Human Research Ethics Committee to recruit people within the Faculty of Medicine and Health Sciences. Participants were not offered any remuneration or gifts to incentivize participation.

### Experimental Design and Tasks

**Figure 1:**
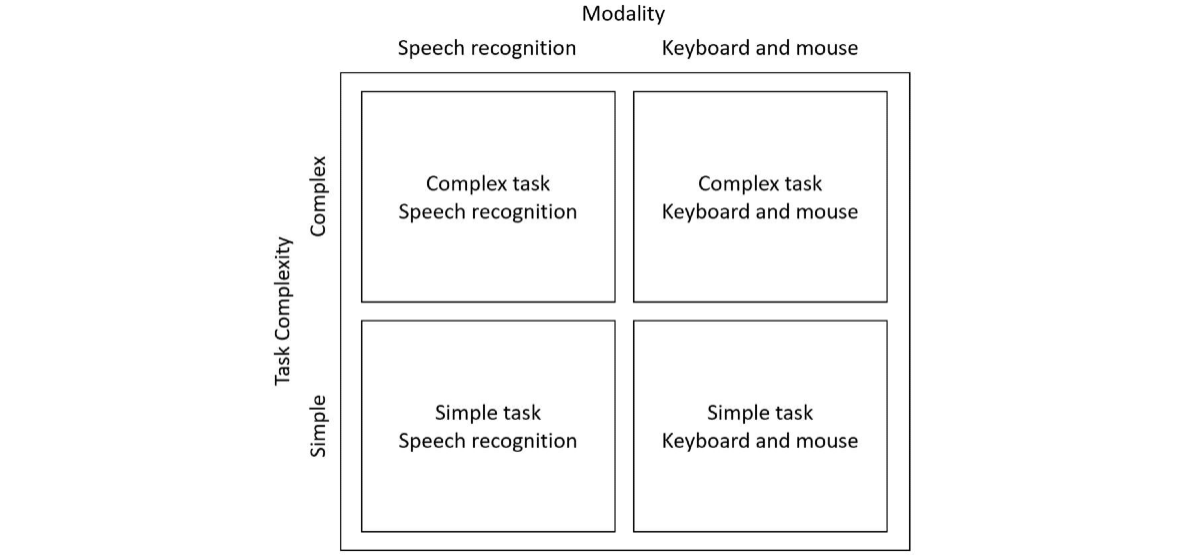
Experimental design.

The study included two within-subject factors: human-computer interaction modality (speech recognition vs keyboard and mouse) and task complexity (simple vs complex) providing four experimental conditions (Figure 1).

Each participant was asked to complete four consumer digital health tasks to document nutritional and exercise information: two simple and two complex tasks, using speech recognition and a keyboard and mouse to interact with a simulated conversational agent (Figure 1). For each documentation task, participants were asked to adopt a persona within a hypothetical scenario focusing on physical activity and diet, and to answer the conversational agent’s questions (see Multimedia Appendix 1). The tasks were designed to assess problem solving and recall using the information provided within the scenario.

Complexity was measured by the number of information items that participants needed to manipulate in working memory. The relationship between the number of information items requiring manipulation in working memory and cognitive load has been well established in the literature [20]; that is, human performance is affected when the cognitive load is too high or exceeds the limits of working memory [21,22]. For simple tasks, the conversational agent displayed the hypothetical scenario on the same screen as the questions. Complex tasks were designed to impose a higher cognitive load by increasing the number of pieces of information needed to perform arithmetic and commit to memory (6 total items for simple tasks vs 17 total items for complex tasks; see Multimedia Appendix 1). Simple tasks required participants to problem solve using 5 items of information, including 5 days, 20 minutes, line dancing, 10:00-10.50 am, whereas complex tasks required participants to recall the same 5 items and were provided with 12 items for problem solving. A higher cognitive load was also imposed by displaying the scenario in a pop-up modal window, requiring participants to commit key information in the scenario to working memory when transferring between the windows [23]. Participants could not copy and paste their answers.

The tasks were developed in consultation with a health informatics researcher (DL) and a primary care physician (LL). Pilot testing was performed by asking 7 individuals to complete the tasks using a prototype of the system and provide feedback. Any issues were iteratively fixed before the next pilot test. This pilot testing ensured that the prescribed tasks and the system were understandable and functional. Individuals who assisted with pilot testing were excluded from participating in the experiment. The correct answers to tasks were predetermined and validated by the health informatics researcher.

### Simulated Conversational Agent

**Figure 2:**
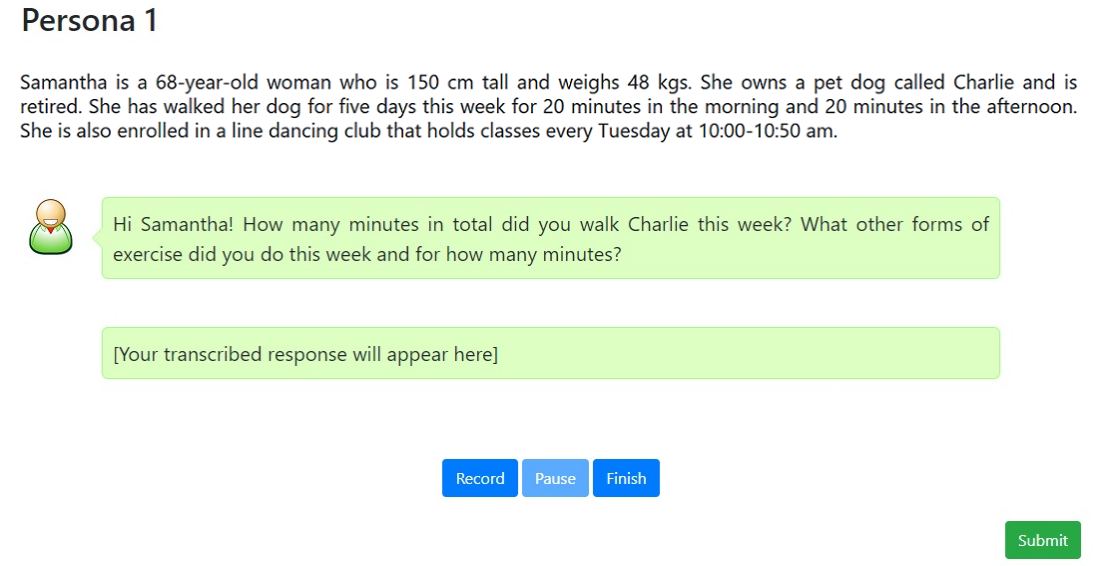
Graphical user interface of the simulated conversational agent on Google Chrome.

Participants attended a computer laboratory at the university where a workstation was set up with the tasks running on a web app connected to a keyboard, mouse, and microphone. Presented as a text message conversation, user responses could either be typed using the keyboard and mouse or transcribed using speech recognition depending on the experimental condition (Figure 2). Participants were able to view their responses before submitting. Web Speech API, a JavaScript-based general purpose speech recognition application programming interface, was used to implement a live audio transcription function for this system [24]. To activate speech-to-text using speech recognition, the participants were instructed to press the “Record” button. The system was launched using Google Chrome on a local machine.

### Procedure

**Figure 3:**
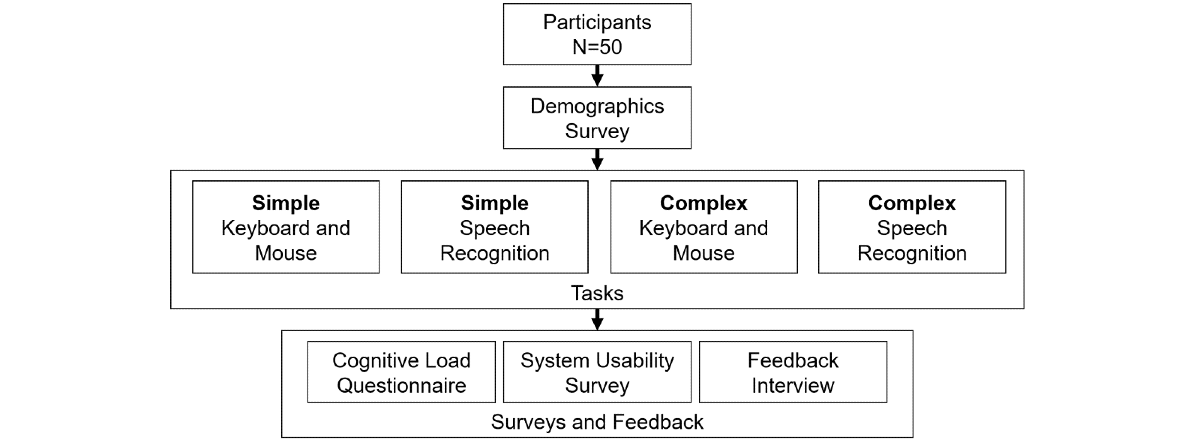
Experimental flow diagram.

After obtaining informed consent, the participants completed a short survey about use of speech recognition technology and provided demographic information. They were then briefed that the tasks consisted of problem solving and recall and given instructions about how to use the speech recognition interface before commencing the experiment. Training continued until the participant clearly understood all aspects required to perform the experimental tasks; no practice tasks were undertaken. Participants completed two simple and two complex tasks, half of which were randomly assigned to using speech recognition (Figure 3). At the end of each task, cognitive load was assessed. To avoid order effects, the assignment of simple and complex tasks for the two modalities and task sequence were randomized. All voice recordings for the speech recognition tasks were captured independently to determine errors. At the end of all four tasks, the System Usability Scale (SUS) questionnaire [25] was completed for each modality followed by a feedback interview.

### Outcome Measures and Analysis

Participants’ responses to the lifestyle management tasks with speech recognition and keyboard and mouse were compared using data extracted from the computer log, audio recordings of participant responses, and paper surveys.

Cognitive load was measured using a cognitive load inventory that was adapted from a validated instrument to reflect the nature of the tasks in the present study [26] (Multimedia Appendix 2). Self-ratings of using this inventory have shown it to be reliable, unobtrusive, and sensitive to small differences [23]. This inventory has been widely used [23,26,27], including in controlled studies of clinical decision making [28]. The inventory was administered on paper at the end of each condition.

Task completion time was measured in seconds and calculated from computer logs as the difference between the task start and end time.

The error rate for a given task was defined as the number of failed recall and problem-solving responses, calculated as a percentage of the maximum score for the task. Participants were asked to answer each of the conversational agent’s questions correctly by recalling information or solving for numerical answers. Each task had predefined correct answers along with numbers associated with correct problem-solving and recall responses (Multimedia Appendix 1). For example, if the correct answers to a task were “line dancing” and “50 minutes,” then the maximum score was 2. If a participant incorrectly recalled “line dancing” but correctly answered “50 minutes,” then the error rate would be calculated as 50%. To ensure that transcription errors were not mistakenly attributed to participants, the voice recording on each response was used to validate the answers for tasks that were completed with speech recognition.

Usability was assessed using the SUS, a validated 10-item questionnaire [25], resulting in overall usability scores and scores of subelements (usability and learnability).

### Statistical Analyses

The effects of speech recognition on cognitive load, task completion time, error rate, and SUS scores were tested using repeated-measures analysis to control for individual differences. The Wilcoxon signed-rank test was used because the results were not normally distributed. We compared speech recognition and keyboard and mouse across both levels of complexity. Cohen criteria were used to calculate and interpret effect sizes (*r*), where 0.1 indicates a small effect, 0.3 indicates a medium effect, and 0.5 indicates a large effect [29]. We estimated that a sample size of 42 was required to detect a difference of 25% and a standard deviation of 20% for each outcome measure for all tasks with 90% power and *P*<.05 [30]. No baseline measures could be derived from the literature. Descriptive statistics were used to summarize demographic information. All statistical analyses were undertaken using SPSS v24.0.0.0 software (IBM Corp, Armonk, NY, USA).

## Results

### Participants

Fifty-two university staff and students participated in the experiment. One participant did not complete the experiment and data from another was lost due to a technical error, leaving a total of 50 participants for inclusion in the analysis. The participants were aged 18-64 years, 30% (15/50) were 25-34 years and 54% (27/50) were women. More than half of the participants (56%, 28/50) reported never using speech recognition in their daily lives, 28% (14/50) reported using it once or twice a week, 14% (7/50) reported using it more than multiple times a week, and 4% (2/50) used speech recognition multiple times a day.

### Effects of Speech Recognition on Cognitive Load

Participants experienced a significantly higher cognitive load when using speech recognition to perform the prescribed tasks (Table 1). These findings were consistent across both levels of task complexity, although the effect size increased from medium for simple tasks to high for complex tasks.

**Table 1:** Comparison of cognitive load between keyboard and mouse and speech recognition use by task complexity.

Task complexity	Keyboard and mouse, median (IQR)	Speech recognition, median (IQR)	*Z* value^a^	*P* value	Effect size (*r*)
Complex	3.2 (2.1-4.2)	5.2 (3.6-6.3)	–4.22	<.001	0.60
Simple	2.2 (2.2-4.4)	3.3 (2.2-5.6)	–2.24	.02	0.32

^a^Wicoxon signed-rank test.

### Task Completion Time and Error Rate

Participants took significantly longer to complete tasks using speech recognition than the keyboard and mouse for complex tasks; however, there was no difference observed for simple tasks (Table 2). For complex tasks, there was a statistically significant increase in task completion time with a medium effect size (*r*=0.36); however, there was no difference for simple tasks.

There was no difference in error rates for both simple and complex tasks (Table 2). For complex tasks, we examined error rates by their type and found no difference for both problem solving (*Z*=–1.96, *P*=.05) and recall error rates (*Z*=–1.55, *P*=.12). 

**Table 2:** Comparison of task completion time and error rates between keyboard and mouse and speech recognition use by task complexity.

Task complexity		Keyboard and mouse	Speech recognition	*Z* value^a^	*P* value
**Complex, median (IQR)**					
	Completion time (seconds)	162 (124-192)	173 (136-223)	2.52	.01
	Error rate (%)	11 (0-25)	11 (0-36)	1.80	.07
**Simple, median (IQR)**					
	Completion time (seconds)	90 (74-124)	83 (68-11)	7.90	.43
	Error rate (%)	0 (0-33)	0 (0-33)	–0.33	.74

^a^Wilcoxon signed-rank test.

### Usability and Participant Perceptions About Speech Recognition

Participants found speech recognition to be significantly less usable than the keyboard and mouse. This was consistent with the SUS factor analysis, which revealed that speech recognition was perceived to be significantly less usable and harder to learn with medium and large effect sizes, respectively (Table 3).

**Table 3:** Usability (SUSa scores) of speech recognition compared to keyboard and mouse.

SUS category	Keyboard and mouse, median (IQR)	Speech recognition, median (IQR)	*Z* value^b^	*P* value	Effect size (*r*)
Overall	85 (72-90)	75 (62-85)	–3.30	.001	0.47
Usability	100 (97-100)	100 (75-100)	–2.98	.003	0.42
Learnability	81 (68-88)	72 (59-84)	–3.54	<.001	0.50

^a^SUS: System Usability Scale.

^b^Wilcoxon signed-rank test.

Overall, participants commented that the simulated conversational agent was “very straightforward” to use. Some reported that not having to think about spelling and grammar was an advantage of speech recognition. Although the participants found speech recognition to be a possibly more convenient way to enter free-text information, many observed that the major pitfalls of speech recognition were any transcription errors generated by the software, an inability to retract and edit answers once a sentence was uttered, and unwanted filler utterances such as “um” and “er” appearing in their responses. Participants also commented about the extra time taken to check and correct the output of speech recognition for transcribing errors when such errors could be prevented by using a keyboard and mouse in the first place. Others raised privacy issues as they were self-conscious about strangers eavesdropping on their responses.

When commenting on difficulties, some participants stated that they had struggled to formulate answers for scenarios and construct sentences to dictate to the conversational agent via speech recognition at the same time. They also commented that this action consumed more time to complete the task. By contrast, typing was perceived to be easier because it was “what we do every day.” This also allowed for using the textbox input to record answers as opposed to “keeping more in the brain” when storing information in cognitive, working memory. Speech was also perceived as a novelty because many were familiar with using a keyboard and mouse and saw it as a standard mode for human-computer interaction.

## Discussion

### Main Findings

When using speech recognition, participants reported a higher cognitive load for both simple and complex tasks compared to using a keyboard and mouse. Some participants reported an inability to think while speaking, which is consistent with previous observations [12,13]. A possible reason is due to an extra step introduced in processing the information and then having to form complete sentences before speaking. This extra processing step, which occurs once participants begin to formulate responses to the simulated conversational agent’s questions, may have involved working memory and contributed to participants experiencing a higher cognitive load while using speech recognition.

The higher cognitive load also provides a potential explanation for the more time required to complete complex tasks when using speech recognition and for it to be perceived less usable. Participants also noted a time delay required for speech recognition to process responses. It is thus possible that the extra time was introduced by the system and the time participants spent in checking the live transcription provided by speech recognition. If this was true, a consistent increase in time across all conditions would be expected. However, there was no significant time difference found for simple tasks. This could mean that the difference more likely arose from the task characteristics themselves. Further experiments are required to examine the interaction effect of modality and task complexity on cognitive load.

Despite previous reports about higher error rates with speech recognition for clinical documentation tasks [31], we found no difference in error rates between the two modalities for both simple and complex tasks. One possible reason that the second hypothesis was not supported is that most participants were observed to formulate their responses with caution by double-checking their answers when using speech recognition. For simple tasks, this meant looking at the scenario section that was on the same screen. For complex tasks, participants repeatedly clicked the “review scenario” button until they were satisfied with their answer.

Speech recognition was perceived to be less usable than a keyboard and mouse because it was harder to learn. A major factor contributing to this effect is that many participants reported being more comfortable with typing because it was an everyday human-computer interaction, especially in their profession. In addition, 56% of the participants did not use speech recognition in their daily lives, which possibly meant that they are less proficient in using speech recognition. Another possible factor is that the conversational agent was purposely designed to limit participants by not allowing users to correct transcription errors. This may be a common source of frustration that affects perceptions of usability for conversational agents. However, the error rate was unaffected because we used independent voice recordings to determine errors and the impact on usability was captured in the overall score.

Some participants reported lack of privacy as another major factor for speech recognition being less usable than a keyboard and mouse. A major advantage with typing was that a third party could not eavesdrop on interactions with the conversational agent. Thus, less confidence was placed on using the conversational agent with speech recognition compared with a keyboard and mouse. Although there was no difference in error rates, the increased cognitive load and task completion time may also have affected user experience.

### Implications

Our findings suggest that speech recognition may not be uniformly suitable for the different contexts of health care. An important implication is for system designers to consider task characteristics and the resulting impact on cognitive load when selecting modalities for humans to interact with computers. In general, lower cognitive load, fewer errors, less time, and better usability are desirable. Speech recognition may be more suitable for frequent tasks such as documentation of notes in electronic health records (EHRs), which may generally not involve problem solving and recall (eg, when a doctor has already established a diagnosis during the patient consultation and uses the EHR to record their notes). However, it may not be suitable for occasional tasks such as incident reporting, which involves problem solving and recall, requiring clinicians to recollect the sequence of events and identify problems that led to an incident [32]. For such complex documentation tasks, the higher cognitive load and greater time required to use speech recognition, along with the lower usability, suggest that a keyboard and mouse may be a better input modality. By contrast, speech recognition may be necessary for contexts that require use of the hands and eyes.

One practical strategy for designers to assess the suitability of speech recognition as an input modality is to test user interfaces in the prototype stage with the cognitive load inventory, which is readily applicable to different modalities and systems. Importantly, designers need to consider privacy requirements when using speech recognition for busy health care settings, especially when sensitive health information is being handled. Our findings also suggest that privacy requirements may present a barrier for the use of speech recognition in some contexts such as health apps that require users to document their personal health information. These considerations will need to be evaluated on a case-by-case basis.

### Comparison With the Literature

To the best of our knowledge, no previous studies have measured the effects of speech recognition in consumer digital health tasks. In clinical applications, the use of speech recognition for clinical documentation was found to increase error rates and task completion time in a controlled laboratory setting [31,33,34]. One possible reason for the disparity with our results may be the difference in the source of complexity. In the previous studies, complexity was distributed between the clinical scenario and the user interactions with an EHR, which required users to navigate to different sections of the record. By contrast, in our study, the type of user interaction remained the same for the different task types. The complexity instead arose from the scenario itself, which required users to problem solve and recall information from memory.

### Limitations

There are several limitations to the design of the current study. We focused on the use of speech recognition as an input modality within a computer laboratory, which may not be representative of a real-world setting where environmental factors such as background noise and interruptions affect consumer interactions with digital health technologies. A general-purpose speech recognition engine that was not specifically optimized for the consumer health domain was tested on a desktop computer. This may have affected participant perceptions about usability and time required to use speech recognition. Participants were university students and staff, almost half of whom reported not using speech recognition technology in their daily lives. Therefore, our sample may not be representative of the general population of health consumers who might use conversational agents. However, because the participants were from a cohort that regularly used a keyboard and mouse, we were able to undertake a realistic assessment of the effort to learn and use speech recognition in the context of the conversational agent. For regular speech recognition users, expectations about the robustness and accuracy could have affected SUS scores. Although it is possible that individual differences such as health literacy, native language, pronunciation, fluency, and experience with speech recognition and a keyboard and mouse may have impacted the outcome variables, we attempted to control for these differences by using a within-subjects design. Further studies are needed to explore the influence of health and nutritional literacy. It is also possible that the quality of the speech recognition could have affected task completion time, but the effect would be consistent across experimental conditions. The error rate was unaffected because we used independent voice recordings to determine correct answers; therefore, the risk of the speech recognition mistranscribing speech by recording responses was controlled.

Despite these limitations, this study has contributed evidence related to the use of speech recognition as an input modality in human-computer interaction, particularly in a consumer digital health context. These results provide baseline measures of cognitive load in using speech recognition. Further studies using a more representative population of conversational agent users are needed to investigate the effects of cognitive load on task performance when speech recognition is integrated with consumer digital health technologies in real-world settings, including mobile devices such as smartphones and tablet computers.

### Conclusions

This study found that using a keyboard and mouse was preferable to speech recognition for complex tasks involving problem solving and recall. This may be due to the higher cognitive load reported when using speech recognition and that the participants were more comfortable using a keyboard and mouse. Our results suggest that task characteristics need to be considered by designers when selecting the most appropriate input modality for human-computer interaction. Further studies using a broader variety of consumer digital health tasks of varying complexity are needed to investigate the contexts in which use of speech recognition is appropriate. The effects of cognitive load on task performance and its significance also need to be investigated.

## Appendix

Multimedia Appendix 1 Tasks.

Multimedia Appendix 2 Cognitive load inventory.

## Abbreviations

EHR: electronic health records
SUS: System Usability Scale
