# Supplementary material for: Effect of Speech Recognition on Problem Solving and Recall in Consumer Digital Health Tasks: Controlled Laboratory Experiment
Source: J Med Internet Res. 2020 Jun 1;22(6):e14827. doi: 10.2196/14827 (PMC7296411; doi:10.2196/14827)
Supplement: Multimedia Appendix 1 [file jmir_v22i6e14827_app1.docx]

**APPENDIX 1: Tasks**

**Scenario A (Simple)**

| Samantha is a 68-year-old woman who is 150 cm tall and weighs 48 kg. She owns a pet dog called Charlie and is retired. She walks her dog five days a week for 20 minutes in the morning and 20 minutes in the afternoon. She is also enrolled in a Line Dancing club that holds classes every Tuesday from 10:00-10:50am.  *Health Buddy question:* *Hi Samantha! How many minutes in total did you walk Charlie this week? What other forms of exercise did you do this week and for how many minutes?* |
| --- |

| Question | Answer |
| --- | --- |
| How many minutes in total did you walk Charlie this week? | 5 days total, 40 minutes per day = 200 minutes |
| What other forms of exercise did you do this week and for how many minutes? | Line dancing for 50 minutes |

Scenario view: On screen
Number of recall items: 0
Number of items used for problem solving: 5
Maximum score: 3
Total items: 6

**Scenario B (Simple)**

| Jason is a 35-year-old man who is 180 cm tall and weighs 75 kg. He works as a Surf Lifeguard on weekends. Last Saturday he rescued five people and on Sunday Jason rescued two people. It would take on average 30 minutes to rescue each person. He also trains for swimming every Wednesday morning from 6:00-6:45am.  *Health Buddy question: Hi Jason! How minutes in total did you spend rescuing people last weekend in your Surf Lifeguard job? What other forms of exercise did you do this week and for how many minutes?* |
| --- |

| Question | Answer |
| --- | --- |
| How minutes in total did you spend rescuing people last weekend in your Surf Lifeguard job? | 7 people total, 30 minutes per person = 210 minutes |
| What other forms of exercise did you do this week and for how many minutes? | Swimming training for 45 minutes |

Scenario view: On screen
Number of recall items: 0
Number of items used for problem solving: 5
Maximum score: 3
Total items: 6

**Scenario C (Complex)**

| Ben is a 21-year-old man who is 170 cm tall and weighs 67 kg. He is part of a state basketball team that plays one competition match each week. This week, his team won their match with a score of 60-40. The night before the match, he cooked for himself a pasta dish consisting of pasta, chicken breast, capsicum and a small can of crushed tomatoes.  *Health Buddy question (part 1): Hi Ben! What was the score of your basketball game this week? Did you have a meal before the match? If so, what did you eat?*  *Health Buddy question (part 2): How many kilojoules, protein, and sodium did you consume?* |
| --- |

|  | **Pasta** | **Chicken breast** | **Capsicum** | **Crushed tomatoes** |
| --- | --- | --- | --- | --- |
| **Energy (kJ)** | 550 | 600 | 20 | 130 |
| **Protein (g)** | 5 | 21 | 0.3 | 1.6 |
| **Fat, total (g)** | 1.1 | 9 | 0 | 0.3 |
| **Saturated (g)** | 0.2 | 2.7 | 0 | 0 |
| **Carbohydrate (g)** | 25 | 0 | 1 | 7 |
| **Sugars (g)** | 0.78 | 0 | 0.6 | 4.4 |
| **Sodium (mg)** | 5 | 65 | 1 | 130 |

| Question | Answer |
| --- | --- |
| What was the score of your basketball game this week? | 60-40 |
| Did you have a meal before the match? If so, what did you eat? | Pasta, chicken breast, capsicum, crushed tomatoes |
| How many kilojoules, protein, and sodium did you consume? | 1200 kilojoules, 27.9 grams of protein, 200 milligrams of sodium |

Scenario view: Hidden, modal window
Number of recall items: 5
Number of items used for problem solving: 12
Maximum score: 9
Total items: 17

**Scenario D (Complex)**

| Michelle is a 24-year-old woman who is 158 cm tall and weighs 56 kg. She is part of a local soccer team that plays a match once a week. Her team lost their match this week with a score of 8-10. After the match she went to McDonald’s and ordered six pieces of nuggets, Sweet ‘n’ Sour sauce, small fries and a regular frozen coke.  *Health Buddy question (part 1): Hi Michelle! What was the score of your soccer game tonight? Did you have a meal after the match? If so, what did you eat?*  *Health Buddy question (part 2): How many kilojoules, protein, and sodium did you consume?* |
| --- |

|  | **Six pieces nuggets** | **Sweet ‘n’ Sour sauce** | **Small fries** | **Frozen coke** |
| --- | --- | --- | --- | --- |
| **Energy (kJ)** | 1100 | 200 | 900 | 700 |
| **Protein (g)** | 14.6 | 0.2 | 3.0 | 0 |
| **Fat, total (g)** | 15.2 | 0.3 | 10.4 | 0 |
| **Saturated (g)** | 2.2 | <0.1 | 0.8 | 0 |
| **Carbohydrate (g)** | 17.1 | 11.2 | 23.5 | 42.6 |
| **Sugars (g)** | 0.5 | 10.5 | 0.1 | 42.6 |
| **Sodium (mg)** | 400 | 120 | 200 | 30 |

| Question | Answer |
| --- | --- |
| What was the score of your soccer game this week? | 8-10 |
| Did you have a meal after the match? If so, what did you eat? | Six pieces of nuggets, sweet ‘n’ sour sauce, small fries, regular frozen coke |
| How many kilojoules, protein, and sodium did you consume? | 2900 kilojoules, 17.8 grams of protein, 750 milligrams of sodium |

Scenario view: Hidden, modal window
Number of recall items: 5
Number of items used for problem solving: 12
Maximum score: 9
Total items: 17
