# Supplementary material for: Effect of Speech Recognition on Problem Solving and Recall in Consumer Digital Health Tasks: Controlled Laboratory Experiment
Source: J Med Internet Res. 2020 Jun 1;22(6):e14827. doi: 10.2196/14827 (PMC7296411; doi:10.2196/14827)
Supplement: Multimedia Appendix 2 [file jmir_v22i6e14827_app2.docx]

**APPENDIX 2: Cognitive Load Inventory**

1. In the task that just finished I invested….

| 1 | 2 | 3 | 4 | 5 | 6 | 7 | 8 | 9 |
| --- | --- | --- | --- | --- | --- | --- | --- | --- |
| Very, very low mental effort |  |  |  | Neither low nor high mental effort |  |  |  | Very, very high mental effort |
